# Correlations among vitamin K intake, body fat, lipid profile and glucose homeostasis in adults and the elderly

**DOI:** 10.20945/2359-3997000000230

**Published:** 2020-03-30

**Authors:** Elizabete A. dos Santos, Kelly V. Giudici, Natasha A. G. de França, Barbara S. Emo Peters, Regina Mara Fisberg, Lígia A. Martini

**Affiliations:** 1 Departamento de Nutrição Faculdade de Saúde Pública Universidade de São Paulo São Paulo SP Brasil Departamento de Nutrição, Faculdade de Saúde Pública, Universidade de São Paulo (USP), São Paulo, SP, Brasil

**Keywords:** Vitamin K intake, phylloquinone, body fat, lipid profile, glucose homeostasis

## Abstract

**Objective:**

Recent research has investigated the possible inverse relationship between vitamin K intake and body fat. In addition, an increasing number of studies are supporting a key role for this vitamin in improving lipid profile and insulin sensitivity and reducing the risk of type 2 diabetes mellitus, but little is known about what mechanisms would be involved. Thus, the objective of this study was to investigate the relationship between vitamin K intake (in the form of phylloquinone – PK), body fat, lipid profile and markers of glucose homeostasis in adults and the elderly.

**Subjects and methods:**

A cross-sectional study with 298 participants (46% men) in the São Paulo Health Survey 2014-2015. Spearman correlations were performed to evaluate the associations between vitamin K intake and the biochemical and body composition measures.

**Results:**

Among normal-weight male adults (n = 15), PK intake presented a positive correlation with the quantitative insulin sensitivity check index (QUICKI) (r = 0.525; p = 0.045). Among men with high fat mass index (FMI) (n = 101), PK intake had a negative correlation with homeostasis model assessment estimate for β-cell function (HOMA-β) (r = −0.227; p = 0.022). In women with high FMI (n = 122), PK intake had a negative correlation with HOMA-β (r = −0.199, p = 0.032) and insulin (r = −0.207, p = 0.026). No correlations were found between PK intake and lipid profile.

**Conclusions:**

Our findings support a potential relationship among PK intake, body fat and markers of glucose homeostasis in adults and the elderly.

## INTRODUCTION

Vitamin K is a fat-soluble nutrient that naturally occurs in two forms: phylloquinone (PK), or vitamin K_1_, and menaquinone (MK), or vitamin K_2_ (1). Phylloquinone is synthesized by plants and is the predominant form of vitamin K found in the human diet. Its main sources are green leafy vegetables (such as cabbage, spinach and broccoli), some fruits (such as avocados, kiwis and green grapes), some herbs (such as parsley, coriander and green tea) and vegetable oils (such as soybean, canola and olive oil) (1,2). Menaquinones include a number of forms of vitamin K, with MK-4 and MK-10 being the major types of MK found in the human diet, especially in foods containing fats, which may increase their absorption and bioavailability compared with PK. Menaquinone may also be endogenously synthesized by the intestinal microbiota of humans and animals, and it can be mainly found in food products with animal origins, such as meat, eggs and cheese (MK-4 and MK-10), and in high amounts in fermented soybean (known as natto) and in legumes (MK-7) (1,2).

Vitamin K was originally recognized for its essential function in blood clotting, acting as a cofactor of the gamma-glutamylcarboxylase (GGCX) enzyme, which converts specific glutamic acid residues of precursor proteins into a new amino acid, gamma carboxyglutamic acid (GLA), found in coagulation factors (3). In addition, other proteins found in blood vessels and bones depend on vitamin K (4), with an increasing number of studies reporting their beneficial effects in reducing the risk of mortality or preventing cardiovascular disease, cancer and bone disorders (5-7).

In addition to its role in blood coagulation, a possible inverse relationship has been studied between vitamin K intake (either through dietary sources or in the form of supplements) and body fat, as well as its metabolic implications, but the findings are not yet conclusive (1,8,9). Since this vitamin is fat-soluble, adipose tissue may physiologically serve as a storage site (9).

Vitamin K intake has also been associated with a favorable lipid profile (1), but research evaluating its possible effects in humans is still scarce. Although their results remain controversial, an increasing number of papers support a key role for this vitamin in improving lipid profile and preventing cardiovascular disease, although little information is available on which mechanisms would be involved (10). Vitamin K, especially in the form of PK, also seems to play an important role in glucose metabolism. In human studies, this vitamin has been shown to have beneficial effects in improving insulin sensitivity and reducing the risk of type 2 diabetes mellitus (DM2) (11-13).

Thus, the objective of the present study was to investigate the relationships among vitamin K intake (in the form of PK), body fat, lipid profile and markers of glucose homeostasis in adults and the elderly.

## SUBJECTS AND METHODS

### Study design and population

The present study was conducted with participants from the São Paulo Municipal Health Survey of 2014-2015 (ISA-Capital 2014-2015), a cross-sectional study carried out through a population-based household survey, in which general information and blood samples were collected from adolescents, adults and elderly residents of the city of São Paulo, Brazil. This cross-sectional study included only adults and elderly residents randomly selected for blood collection. Pregnant women were not included. ISA-Capital 2014-2015 was approved by the Research Ethics Committee (REC) of the School of Public Health of the University of São Paulo (FSP/USP) under protocol CAAE 44552815.0.0000.5421. All of the participants signed an informed consent form.

### Anthropometric measurements

Weight was measured with a Tanita^®^ portable digital scale, with a maximum capacity of 150 kg and a precision of 0.1 kg, with barefoot individuals wearing light clothing and no accessories, such as a watch or necklaces. Height was measured with a Seca^®^ wall stadiometer, with a precision of 0.1 cm. The measurements were taken in duplicate, non-consecutively, and a third measurement was taken when a difference was greater than 0.1 kg or 0.5 cm between the first and second values. In this case, the average of the two closest measurements was used, and the other measure was discarded. Subsequently, the body mass index (BMI) was calculated according to the equation proposed by Quetelet (14): [BMI (kg/m^2^) = Weight (kg) / Height (m) x Height (m).

Waist (WC) and hip circumferences (HC) were measured in duplicate with an inelastic anthropometric tape, with an accuracy of 0.1 cm. A third measurement was necessary when the difference between the first two was greater than 1 cm. In this case, the average of the two closest measures was used, and the other measure was discarded. The WC was taken at the midpoint between the last intercostal arch and the iliac crest. The HC was measured in the region with the highest protuberance and was used to calculate the waist-to-hip ratio.

Fat mass was quantitatively investigated using dual-energy X-ray absorptiometry (DXA) (iDXA Advance model, GE Healthcare, Madison, WI, USA), with individuals supinely positioned on the appliance table and wearing light clothing (or an apron) and an elastic band for the breast region (for women), without any metallic adornment. Values of total body fat, percentages of android and gynoid fat, android/gynoid rate and visceral adipose tissue were determined. To evaluate excess adiposity, absolute values of total fat mass were considered, and the fat mass index (FMI) was calculated according to Kelly and cols. (15) as: total body fat (kg) / height (m^2^). Excessive body fat was defined as FMI > 6.01 kg/m^2^ for men and > 9.01 kg/m^2^ for women (15).

### Vitamin K intake

Food intake data were collected through two 24-hour food recalls (R24h). The first was collected personally at participants’ homes by a trained interviewer, and a second R24h was collected after 185 days (on average) in 75% of the sample through telephone interviews. The second R24h was collected on a different weekday on a non-consecutive day, which allowed for correcting the intrapersonal variance in diets. At the time of the first R24h, the subject’s use of any dietary supplements containing vitamin K was also checked.

Food intake was recorded through home measurements considering the preparation mode, ingredients and trademarks of industrialized products, following the procedures described in the multiple pass method (MPM), developed by the US Department of Agriculture (USDA). The home measurements were converted into units of weight (g) and volume (mL) according to standardizations described by Pinheiro and cols. (16) and Fisberg and Villar (17). This conversion is a necessary step for using the Nutrition Data System for Research (NDS-R) software (version 2007, Nutrition Coordinating Center, University of Minnesota, Minneapolis, USA), which incorporates MPM procedures. When information on regional foods and preparations was absent, the nutritional value was estimated based on the national food composition table (Brazilian Food Composition Table – TACO) (18). As there is still insufficient data to establish the estimated average requirement (EAR) values for vitamin K, determining inadequate vitamin K intake is impossible. The Food and Nutrition Board of the Institute of Medicine (FNB/IOM) (19) established adequate intake (AI) values for adults from consumption data of a healthy representative sample of the North American population. In this study, AI values (90 μg for women and 120 μg for men) were used as cutoff points to create subgroups of participants with low dietary intake of vitamin K.

### Biochemical variables

Blood samples were collected after 12 to14 hours of fasting and sent to the Laboratory of Nutritional Genomics and Inflammation of the FSP/USP for processing and storage. All biochemical measurements were taken in a commercial laboratory. Lipid profile was determined with serum concentrations of total cholesterol (TC), low-density lipoprotein cholesterol (LDL-c), high-density lipoprotein cholesterol (HDL-c) and triacylglycerols (TG), measured by colorimetric enzymatic methods (Cobas-Roche Diagnostics GmbH, Mannheim, Germany). The concentration of very low density lipoprotein cholesterol (VLDL-c) was obtained according to the following formula: VLDL-c (mg/dL) = TG (mg/dL) / 5 (20). Lipid profile was considered altered when TC ≥ 190 mg/dL; HDL-c < 40 mg/dL, LDL-c ≥ 100 mg/dL; VLDL-c > 30 mg/dL or TG ≥ 150 mg/dL (21).

Fasting glycemia was determined using the enzymatic colorimetric method for glucose oxidase (Trinder’s reaction; Labtest Diagnóstica^®^; Lagoa Santa, MG, Brazil), and serum insulin was evaluated by multiplex immunoassay (LINCOplex^®^; Linco Research Inc., St. Charles, MO, USA). The homeostasis model assessment estimate for insulin resistance (HOMA-IR) and homeostasis model assessment estimate for β-cell function (HOMA-β) were calculated according to the formulas proposed by Matthews and cols. (22): HOMA-IR = fasting glucose (mg/dL)] x [fasting insulin (mIU/L)] / 405; and HOMA-β = 360 x [fasting insulin (mIU/L)] / [fasting glucose mg/dL)] − 63. The quantitative insulin sensitivity check index (QUICKI) was calculated according to the formula proposed by Katz and cols. (23): 1 / (log fasting insulin + log fasting glucose). Blood glucose was considered altered when ≥ 100 mg/dL (24). The presence of insulin resistance was defined as when HOMA-IR > 4.65 or when HOMA-IR > 3.60 accompanied by BMI > 27.5 kg/m^2^ (25).

### Statistical analysis

A descriptive statistical analysis was performed, with data presented as medians, interquartile ranges and frequencies (%). The distributions of all of the continuous variables were evaluated using the Shapiro-Wilk test. Vitamin K had its intrapersonal variability adjusted by the Multiple Source Method (MSM) program, an online program developed by the Department of Epidemiology of the German Institute of Human Nutrition Postdam-Rehbrucke (DIfE) that uses R24h (among other types of food surveys) in a statistical modeling technique to estimate the habitual intake of repeated measurements of this instrument (26). In order to minimize errors due to over-reporting or sub-reporting of intake, R24h with energy values higher than 4,000 kilocalories or lower than 500 kilocalories were excluded. For all statistical analyses, energy-adjusted vitamin K was used according to the formula (vitamin K / total energy intake) x 1,000.

Since none of the variables presented a normal distribution, Spearman’s correlation test was performed. Then, for characterization, individuals with vitamin K intake below 90 μg (for women) or 120 μg (for men) were divided into groups according to sex, age and nutritional status (defined by BMI) or fat mass index classification. Differences between median vitamin K intake according to age, sex and schooling were evaluated using the Mann-Whitney-Wilcoxon (rank-sum) and Kruskal-Wallis tests.

The Stata software, developed by StataCorp LP^®^ was used to perform the analyses, and a significance level of 5% (α = 0.05) was considered for all of the statistical tests. Therefore, the results with p < 0.05 were considered statistically significant.

## RESULTS

A total of 298 individuals were evaluated, of whom 46% were male (n = 136), with a median age of 61 years (Table 1). Among the sample, 56.4% were overweight or obese, and obesity was more prevalent among elderly compared to adult participants (62.8% vs. 37.2%; p = 0.0345). Among the total sample, 75.2% (n = 224) presented high FMI, with no difference according to sex or age. Changes in at least one of the evaluated metabolic parameters were observed in 264 individuals (88.6%), with the main ones being high LDL-c serum (65%) and high serum glucose (48.1%); changes was higher among men (p = 0.0340).

**Table 1:** Anthropometric, biochemical and nutrient intake characteristics of the study participants, according to sex

	Total (n = 298) Median (Min-Max)	Male (n = 136) Median (Min-Max)	Female (n = 162) Median (Min-Max)	p
Age (Years)	61 (20-94)	61,5 (20-94)	60 (20-84)*	0.029
Adults	48 (20-59)	49 (20-59)	46 (20-59)	0.701
Elderly	66 (60-94)	67 (60-94)	66 (60-84)	0.230
**Measures of body composition**				
Weight (kg)	71.9 (35.1-125.1)	77.5 (42.4-123.3)	68 (35.1-125.2)**	0.0000
Height (m)	1.63 (1.40-1.9)	1.70 (1.48-1.89)	1.58 (1.40-1.78)**	0.0000
BMI (kg/m^2^)	27.5 (16.7-50.3)	27.2 (17.5-38.5)	28 (16.7-50.3)	0.064
WC (cm) (n = 296)	94.7 (0.6-141.5)	96.7 (66.8-132.8)	93 (6-141.5)*	0.006
HC (cm) (n = 296)	101.8 (81.5-152.5)	101 (81.5- 128.9)	102.5 (81.5-152.5)	0.016
W/H ratio (n = 296)	0.92 (0.05-1.17)	0.96 (0.75-1.17)	0.90 (0.05-1.12)**	0.0000
Total body fat (%)	36.0 (8.0-55.7)	29.9 (8-52.3)	40.9 (11.6-55.7)**	0.0000
Total body fat (kg)	25.2 (4.0-67.1)	22.6 (4.7-58)	27.7 (4.03-67.1)**	0.0000
Android fat (%)	43.4 (4.3-65.9)	39.4 (4.3-60.7)	47.7 (4.7-65.9)**	0.0000
Gynoid fat (%)	39.2 (6.9-60.4)	29.3 (6.9-54.6)	44.6 (9.9-60.4)**	0.0000
A/G rate	1.1 (0.5-2.1)	1.3 (0.5-2.1)	1.1 (0.5-1.5)**	0.0000
FMI (kg/m^2^)	9.6 (1.54-47.4)	7.9 (1.5-18.9)	11.3 (1.92-47.4)**	0.0000
Visceral Adipose Tissue (cm^3^)	1222.5 (1.5-4722.0)	1629 (6.0-4722.0)	1064,0 (1.5-3963.0)**	0.0000
Visceral Adipose Tissue (g)	1161.0 (1.4-4455.0)	1523.5 (6-4455)	1014.0 (1.4-3739.0)**	0.0000
**Markers of glucose homeostasis**				
Insulin (mUI/mL) (n = 296)	10.2 (1.9-55.4)	9.95 (2-45.3)	10.9 (1.9-55.4)	0.399
Blood glucose (mg/dL) (n = 297)	99 (74-346)	101 (76-346)	97 (74-242)*	0.034
HOMA-IR (n = 296)	2.5 (0.4-25.2)	2.6 (0.42-16.2)	2.49 (0.37-25.2)	0.929
HOMA-β (n = 296)	110 (3.8-628.3)	101.3 (3.8-628.3)	116.4 (16.9-588.3)*	0.017
QUICKI (n = 296)	0.33 (0.25-0.46)	0.33 (0.26-0.45)	0.33 (0.25-0.46)	0.929
**Lipid profile**				
TC (mg/dL) (n = 289)	183 (82-450)	182 (82-313)	184.5 (97-450)	0.686
HDL-c (mg/dL) (n = 289)	47 (10-94)	46 (10-87)	47,5 (14-94)	0.765
LDL-c (mg/dL) (n = 289)	113 (28-373)	112 (28-204)	113 (36-373)	0.515
VLDL-c (mg/dL) (n = 289)	23 (6-113)	23 (7-113)	22 (6-60)	0.072
No-HDL- c (mg/dL) (n = 289)	140 (49-392)	144 (52-276)	137 (49-392)	0.659
TG (mg/dL) (n = 289)	114 (32-567)	115 (33-567)	111.5 (32-300)	0.078
**Nutrient Intake**	1763.1			
Energy (kcal) (n = 292)	1597.2 (505-3980.6)	1763.1 (505-3980.6)	1462.9 (599-3738.5)**	0.0000
Vitamin K (μg) (n = 292)	102.7 (56.7-191.8)	102.4 (56.7-156.5)	103 (57.3-191.8)	0.752
Vitamin K (μg/1,000 kcal) (n = 292)	60.1 (33.2-112.2)	59.9 (33.2-91.5)	60.2 (33.5-112.2)	0.752

BMI: body mass index; WC: waist circumference; HC: hip circumference; W/H ratio: waist-hip ratio; A/G rate: android-gynoid rate; FMI: Fat Mass Index; HOMA-IR: Homeostasis Model Assessment Estimate for Insulin Resistance; HOMA-β: Homeostasis Model Assessment Estimate for β Cell Function; QUICKI: Quantitative Insulin Sensitivity Check Index; TC: Total Cholesterol; HDL-c: High Density Lipoprotein Cholesterol; LDL-c: Low Density Lipoprotein Cholesterol; VLDL-c: Very Low Density Lipoprotein Cholesterol; Non-HDL-c: non-HDL cholesterol; TG: Triacylglycerol. * p < 0.05 (Mann-Whitney Test). ** p < 0.0001 (Mann-Whitney Test).

The median vitamin K intake was 102.7 μg (or 60.1 μg/1,000 kcal of diet), with no differences according to sex or age. Considering AI values according to sex, 78% (n = 101) of men and 25% (n = 40) of women presented vitamin K intake lower than 120 μg or 90 μg, respectively.

**Figure 1:**
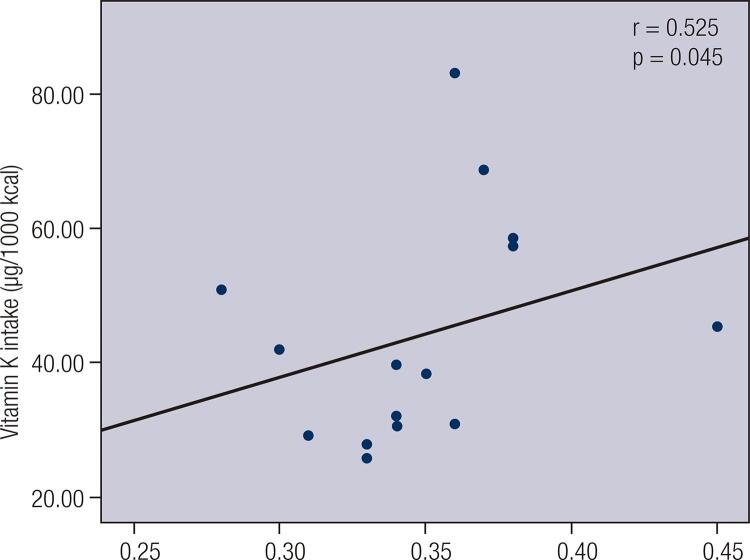
Spearman’s correlation between vitamin K intake and quantitative insulin sensitivity check index (QUICKI) among normal weight male adults with vitamin K intake lower than 120 μg.

**Figure 2:**
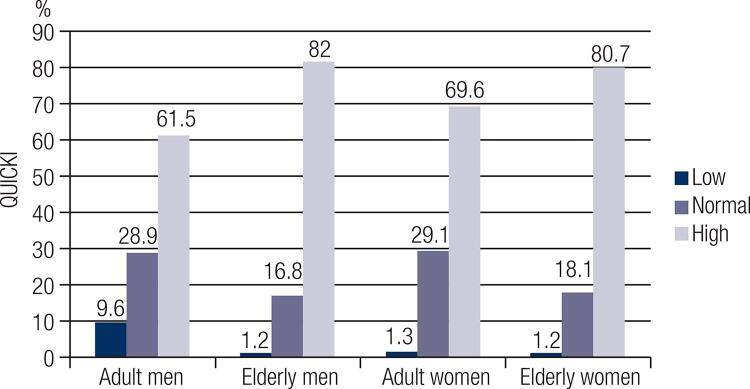
Percentage of men and women, according to fat mass index and life stage obtained by DXA.

**Figure 3:**
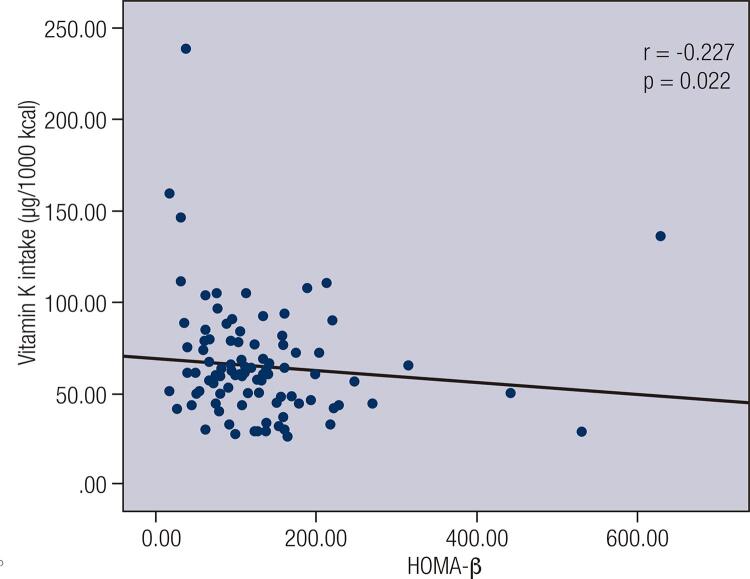
Spearman's correlation between vitamin K intake and homeostasis Homeostais model assessment estimate for β cell function (HOMA-β) among adult and elderly men with high fat mass index (FMI).

**Figure 4:**
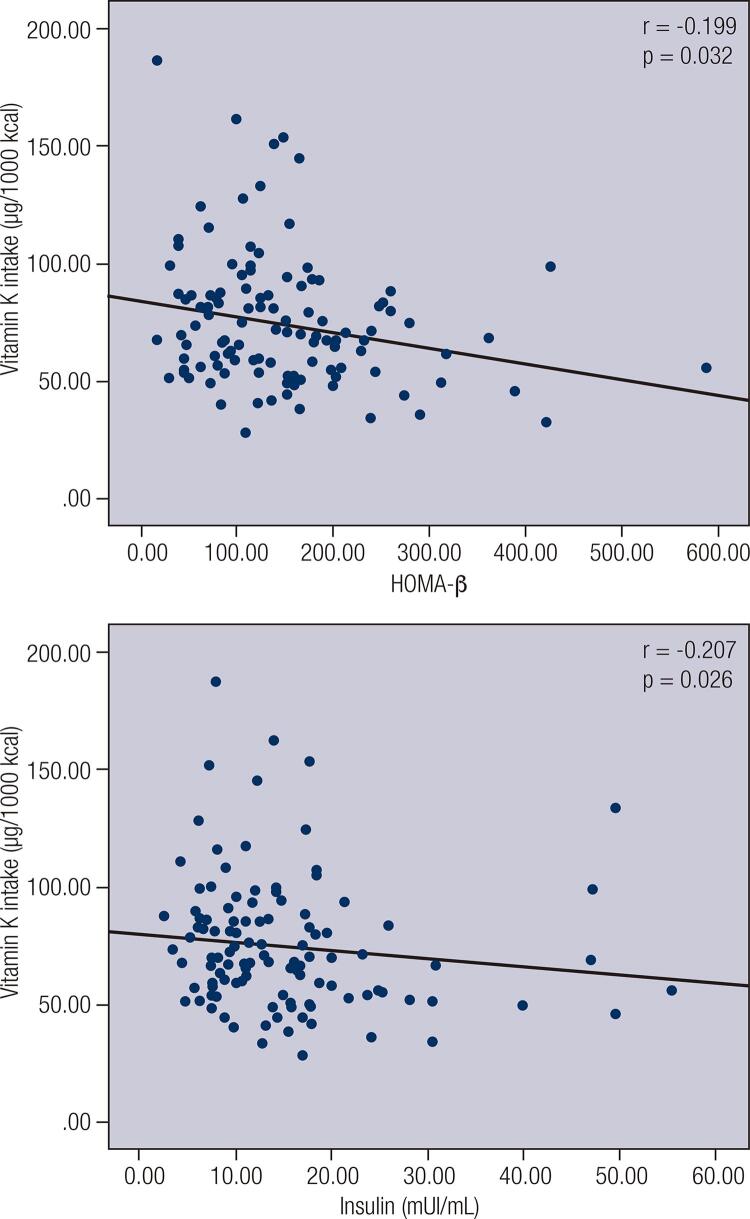
Spearman's correlation between vitamin K intake and homeostasis Homeostasis model assessment estimate for β cell function (HOMA-β) and between vitamin K intake and Insulin among adult and elderly women with high fat mass index (FMI).

A negative correlation between vitamin K intake and blood glucose (r = −0.1799; p = 0.0224), a negative correlation between vitamin K intake and HOMA–IR (r = −0.1617; p = 0.0411) and a positive correlation between vitamin K intake and QUICKI (r = 0.1617; p = 0.0411) were observed among women (n = 160) (Table 2).When individuals with low vitamin K intake were divided into subgroups according to nutritional status and age, a positive correlation between vitamin K intake and QUICKI (r = 0.525; p = 0.045) was observed among normal-weight male adults (n = 15) (Figure 1). When individuals were divided into subgroups according to FMI and sex (Figure 2), vitamin K intake had a negative correlation with HOMA-β (r = −0.227; p = 0.022) among men with high FMI (n = 101) (Figure 3), and vitamin K intake had a negative correlation with HOMA-β (r = −0.199, p = 0.032) and insulin (r = −0.207, p = 0.026) among women with high FMI (n = 122) (Figure 4). No correlations were found between vitamin K intake and lipid profile.

**Table 2:** Spearman's correlation for measures of body composition and metabolic parameters according to sex

	Total (n = 298)		Male (n = 136)		Female (n = 162)	
		
	r	p	r	p	r	p
**Measures of body composition**						
Weight (kg)	-0.0410	0.4803	-0.0348	0.6876	-0.0373	0.6371
Height (m)	-0.0773	0.1833	-0.0801	0.3537	--0.1112	0.1590
BMI (kg/m^2^)	-0.0074	0.8988	-0.0338	0.6957	0.0046	0.9535
WC (cm) (n = 296)	0.0020	0.9724	0.0107	0.9026	-0.0055	0.9448
HC (cm) (n = 296)	-0.0071	0.9033	-0.0244	0.7796	-0.0070	0.9293
W/H ratio (n = 296)	0.0115	0.8438	0.0379	0.6641	0.0037	0.9628
Total body fat (%)	0.0078	0.8933	-0.0237	0.7839	0.0278	0.7251
Total body fat (kg)	-0.0109	0.8509	-0.0360	0.6774	0.0039	0.9605
Android fat (%)	0.0105	0.8568	-0.0041	0.9618	0.0315	0.6910
Gynoid fat (%)	-0.0011	0.9846	-0.0337	0.6967	0.0012	0.9876
A/G rate	0.0000	0.9993	0.0545	0.5289	-0.0399	0.6139
FMI (kg/m^2^)	-0.0052	0.9286	-0.0346	0.6890	0.0100	0.8992
Visceral Adipose Tissue (cm^3^)	0.0067	0.9088	-0.0094	0.9133	0.0200	0.8004
Visceral Adipose Tissue (g)	-0.0016	0.9776	-0.0001	0.9992	-0.0073	0.9262
**Metabolic parameters**						
Insulin (mUI/mL) (n = 296)	-0.0728	0.2119	-0.0106	0.9024	-0.1314	0.0978
Blood glucose (mg/dL) (n = 297)	-0.1103	0.0577	-0.0195	0.8220	-0.1799*	0.0224
HOMA-IR (n = 296)	-0.0685	0.2403	0.0337	0.6965	-0.1617*	0.0411
HOMA-β (n = 296)	-0.0101	0.8629	-0.0274	0.7519	-0.0040	0.9602
QUICKI (n = 296)	0.0685	0.2403	-0.0337	0.6965	0.1617*	0.0411
TC (mg/dL) (n = 289)	-0.0289	0.6252	-0.0205	0.8147	-0.0327	0.6852
HDL-c (mg/dL) (n = 289)	0.0608	0.3028	0.0754	0.3881	0.0369	0.6470
LDL-c (mg/dL) (n = 289)	-0.0273	0.6445	-0.0267	0.7601	-0.0327	0.6856
VLDL-c (mg/dL) (n = 289)	0.0381	0.5191	-0.0285	0.7448	0.1013	0.2083
No-HDL- c (mg/dL) (n = 289)	-0.0149	0.8005	-0.0186	0.8321	-0.0192	0.8117
TG (mg/dL) (n = 289)	0.0379	0.5209	-0.0308	0.7247	0.1036	0.1982

BMI: body mass index; WC: waist circumference; HC: hip circumference; W/H ratio: waist-hip ratio; A/G rate: android-gynoid rate; FMI: Fat Mass Index; HOMA-IR: Homeostasis Model Assessment Estimate for Insulin Resistance; HOMA-β: Homeostasis Model Assessment Estimate for β Cell Function; QUICKI: Quantitative Insulin Sensitivity Check Index; TC: Total Cholesterol; HDL-c: High Density Lipoprotein Cholesterol; LDL-c: Low Density Lipoprotein Cholesterol; VLDL-c: Very Low Density Lipoprotein Cholesterol; Non-HDL-c: non-HDL cholesterol; TG: Triacylglycerol. * p < 0.05 (Spearman Correlation Test).

## DISCUSSION

In the present study to evaluate the relationships among vitamin K intake, body fat, lipid profile and glucose homeostasis among adults and the elderly, vitamin K intake was positively associated with better insulin sensitivity among women and normal-weight adult men with vitamin K intake below 120 μg. Among women, vitamin K intake had a negative correlation with HOMA-IR and blood glucose, and among men and women with high FMI, vitamin K intake had a negative correlation with HOMA-β and, among only women, with insulin. Evaluating men and women aged 26 to 81 years, Yoshida and cols. (27) observed that PK intake, evaluated by a food frequency questionnaire (FFQ), was not associated with HOMA-IR concentrations or with fasting insulin, glucose or glycated hemoglobin concentrations (HbA1C). However, the same investigators found that in elderly men, daily supplementation of 0.5 mg of PK for 36 months had a protective effect on the progression of insulin resistance, defined by HOMA-IR (10). Methodological differences regarding the form of vitamin K intake (diet vs. supplementation), its type (PK vs. MK) and the instrument used to evaluate food intake (R24h vs. FFQ) may have contributed to the discrepancies in the results.

In any case, vitamin K is known to act as a cofactor in the carboxylation of some proteins, such as growth-arrest-specific gene 6 (GAS6), osteocalcin (OC), prothrombin and protein S, which have also had an identified role in glucose homeostasis in important organs such as the liver and pancreas, which could be a possible biological mechanism to explain this association (10,28,29). Since inflammation is present in chronic metabolic conditions, the effects of vitamin K on glucose homeostasis may also occur by vitamin K modulating cytokines and metabolic risk markers related to insulin resistance and DM2 (11). Ohsaki and cols. (30) observed that vitamin K treatment in cultured cells reduced the expression of interleukin 6 (IL-6). The authors stated that the anti-inflammatory effect of vitamin K could be expressed through reduced oxidative stress and/or inhibition of the nuclear factor kappa-light-chain-enhancer of activated B cells (NFkβ), and thus inhibit the production of pro-inflammatory cytokines.

Therefore, the anti-inflammatory effects of vitamin K may also be related to a favorable lipid profile. As previously stated, vitamin K plays an important role in controlling inflammation by inhibiting the production of pro-inflammatory mediators, such as TNF and CRP, which, when increased in circulation, may contribute to an atherogenic lipid profile (10,31). However, no correlation was found between vitamin K intake and lipid profile in the present study. Kolahi and cols. (12) also did not observe changes in lipid profile in their study of vitamin K supplementation (10 mg of PK/day) for 8 weeks in women with rheumatoid arthritis. In contrast, Thane and cols. (32) found that plasma PK concentrations were significantly associated with plasma TC and LDL-c concentrations in individuals aged 19 to 64 years. Notably, the populations of these studies have differing sex, nutritional status and age range, which may interfere with comparing their findings with those of the present study.

Among men and women with elevated FMI, vitamin K intake was negatively correlated with markers of glucose metabolism. As this was not found in individuals with normal levels of body fat, fat mass may have influenced this result. Because vitamin K is a lipid-soluble vitamin, the amount and availability of vitamin K in the tissues may vary by the amount of body fat, which means that individuals with higher adiposity may be at greater risk of vitamin K insufficiency (9). Indeed, Shea and cols. (9) showed that elderly women with higher body fat had lower plasma vitamin K (PK) concentrations and higher levels of protein induced by vitamin K absence-II (PIVKA-II). High concentrations of PIVKA-II were found in older men with higher body fat percentages, suggesting that increased adiposity is associated with lower levels of vitamin K. These findings are consistent with those of a study by Sogabe and cols. (1), in which rats consuming a diet with added PK showed significantly reduced visceral fat, whereas those with added MK showed significantly reduced visceral and subcutaneous fat.

A possible explanation for the effects of vitamin K on body weight is that it acts directly on cellular functions, which are independent of gamma-carboxylation (2). Takeuchi and cols. (33) suggested that MK-4 specifically influences the differentiation and functions of bone marrow cells to inhibit adipogenesis and osteoclastogenesis. In vitro studies performed by these authors provide evidence that MK-4 treatment inhibits adipogenesis, the expression of the osteoclast differentiation factor (ODF)/receptor activator of nuclear factor*-*kappa*-*B-ligand (RANKL) and the formation of osteoclast-like cells induced by 1,25-dihydroxyvitamin D_3_.

Note that in the present study, more than half of the evaluated men and 25% of the evaluated women presented low dietary intake of vitamin K. However, according to the recommendation of the Dietary Reference Intakes (DRI) Committee, no inferences can be made about the adequacy or inadequacy of intake when a nutrient only presents AI values since the individual’s need for the nutrient is unknown. In addition, alterations in at least one of the evaluated metabolic parameters were observed in 88.6% of the sample, especially high serum LDL-c and high serum glucose.

An estimated 40% of Brazilians have hypercholesterolemia (34); in addition, global estimates from the WHO (35) point out that 422 million adults were living with diabetes in 2014 and that higher-than-optimal blood glucose caused an additional 2.2 million deaths, by increasing the risks of cardiovascular and other diseases. In this sense, the investigations of the present study are relevant for creating strategies to emphasize the importance of a balanced and healthy diet containing all essential micronutrients, such as vitamin K.

Importantly, all necessary care was taken to evaluate vitamin K intake in our study. The use of two R24h measurements was a positive factor, as is the exclusion of R24h measurements with energy consumption below 500 kcal and above 4,000 kcal, which minimized possible under- or over-reporting errors. In addition, energy-adjusted vitamin K was used in all of the statistical analyses, and its intrapersonal variability was adjusted by MSM. Body fat was assessed using DXA, which is considered the gold standard in evaluating body composition, and other anthropometric measures were also taken to allow a better evaluation of body adiposity.

On the other hand, some limitations must also be mentioned, such as the evaluation of PK but not of MK. However, the current recommendations are based on databases containing the nutritional compositions of foods, which only include PK content. Moreover, due to its observational cross-sectional design, it is not possible to infer causality, making it difficult to compare the results with those of longitudinal studies. Evaluating inflammatory markers and plasma PK concentrations also could have enriched the investigation.

In conclusion, our findings support the existence of a relationship among vitamin K intake (in the form of PK), body fat and markers of glucose homeostasis in adults and the elderly. These results reinforce the need for more studies and greater attention to vitamin K intake, given that this vitamin can be a marker of good food quality in the context of a healthy diet. Nutritional strategies, including recommendations for healthy food intake, such as of vitamin K sources, may help to prevent excessive accumulation of body fat and metabolic alterations such as hyperglycemia, considering that these conditions are related to the emergence of chronic non-communicable diseases, especially cardiovascular disease.

## References

[1] Sogabe N, Maruyama R, Baba O, Hosoi T, Goseki-Sone M. Effects of long-term vitamin K(1) (phylloquinone) or vitamin K(2) (menaquinone-4) supplementation on body composition and serum parameters in rats. Bone. 011;48(5):1036-42. 10.1016/j.bone.2011.01.020 21295170

[2] Knapen MHJ, Schurgers LJ, Shearer MJ, Newman P, Theuwissen E, Vermeer C. Association of vitamin K status with adiponectin and body composition in healthy subjects: uncarboxylated osteocalcin is not associated with fat mass and body weight. Br J Nutr. 2012;108(6):1017-24. 10.1017/S000711451100626X 22136751

[3] Iwamoto J, Takada T, Sato Y. Vitamin K nutritional status and undercarboxylated osteocalcin in postmenopausal osteoporotic women treated with bisphosphonates. Asia Pac J Clin Nutr. 2014;23(2):256-62. 10.6133/apjcn.2014.23.2.15 24901095

[4] Yamauchi M, Yamaguchi T, Nawata K, Takaoka S, Sugimoto T. Relationships between undercarboxylated osteocalcin and vitamin K intakes, bone turnover, and bone mineral density in healthy women. Clin Nutr. 2010;29(6):761-5. 10.1016/j.clnu.2010.02.010 20332058

[5] Hao G, Zhang B, Gu M, Chen C, Zhang K, Zhang G, et al. Vitamin K intake and the risk of fractures: A meta-analysis. Medicine (Baltimore). 2017;96(17):e6725. 10.1097/MD.0000000000006725 PMC5413254 28445289

[6] Juanola-Falgarona M, Salas-Salvadó J, Martínez-González MÁ, Corella D, Estruch R, Ros E, et al. Dietary intake of vitamin K is inversely associated with mortality risk. J Nutr. 2014;144(5):743-50. 10.3945/jn.113.187740 24647393

[7] Shea MK, Booth SL, Weiner DE, Brinkley TE, Kanaya AM, Murphy RA, et al. Circulating Vitamin K is Inversely Associated with Incident Cardiovascular Disease Risk among Those Treated for Hypertension in the Health, Aging, and Body Composition Study (Health ABC). J Nutr. 2017;147(5):888-95. 10.3945/jn.117.249375 PMC5404216 28356433

[8] Knapen MHJ, Jardon KM, Vermeer C. Vitamin K-induced effects on body fat and weight: results from a 3-year vitamin K2 intervention study. Eur J Clin Nutr. 2018;72(1):136-141. 10.1038/ejcn.2017.146 28952607

[9] Shea MK, Booth SL, Gundberg CM, Peterson JW, Waddell C, Dawson-Hughes B, et al. Adulthood Obesity Is Positively Associated with Adipose Tissue Concentrations of Vitamin K and Inversely Associated with Circulating Indicators of Vitamin K Status in Men and Women. J Nutr. 2010;140(5):1029-34. 10.3945/jn.109.118380 PMC2855266 20237066

[10] Kolahi S, Pourghassem Gargari B, Mesgari Abbasi M, Asghari Jafarabadi M, Ghamarzad Shishavan N. Effects of phylloquinone supplementation on lipid profile in women with rheumatoid arthritis: a double blind placebo controlled study. Nutr Res Pract. 2015;9(2):186-91. 10.4162/nrp.2015.9.2.186 PMC4388951 25861426

[11] Yoshida M, Jacques PF, Meigs JB, Saltzman E, Shea MK, Gundberg C, et al. Effect of Vitamin K Supplementation on Insulin Resistance in Older Men and Women. Diabetes Care. 2008;31(11):2092-6. 10.2337/dc08-1204 PMC2571052 18697901

[12] Juanola-Falgarona M, Salas-Salvadó J, Estruch R, Portillo MP, Casas R, Miranda J, et al. Association between dietary phylloquinone intake and peripheral metabolic risk markers related to insulin resistance and diabetes in elderly subjects at high cardiovascular risk. Cardiovasc Diabetol. 2013;12:7. 10.1186/1475-2840-12-7 PMC3558443 23298335

[13] Manna P, Kalita J. Beneficial role of vitamin K supplementation on insulin sensitivity, glucose metabolism, and the reduced risk of type 2 diabetes: A review. Nutrition. 2016;32(7-8):732-9. 10.1016/j.nut.2016.01.011 27133809

[14] Quételet A. Antropométrie ou mesure desdifférentes facultés de l’homme. Bruxelles: C. Muquardt; 1870.

[15] Kelly TL, Wilson KE, Heymsfield SB. Dual energy X-Ray absorptiometry body composition reference values from NHANES. PLoS One. 2009;4(9):e7038. 10.1371/journal.pone.0007038 PMC2737140 19753111

[16] Pinheiro AB, Lacerda EMA, Benzecry EH, Gomes MCS, Costa VM. Tabela para avaliação de consumo alimentar em medidas caseiras. São Paulo: Atheneu; 2004.

[17] Fisberg RM, Villar BS. Manual de receitas e medidas caseiras para cálculo de inquéritos alimentares: manual elaborado para auxiliar o processamento de dados de inquéritos alimentares. São Paulo: Signus; 2002.

[18] Tabela brasileira de composição de alimentos/Nepa-Unicamp. Campinas: Nepa-Unicamp; 2011.

[19] Food and Nutrition Board, Institute of Medicine. Dietary Reference Intakes for Vitamin A, Vitamin K, Arsenic, Boron, Chromium, Copper, Iodine, Iron, Manganese, Molybdenum, Nickel, Silicon, Vanadium, and Zinc. Washington, DC, USA: National Academy Press; 2002.

[20] Fisberg RM, Sales CH, Fontanelli MM, Pereira JL, Alves MCGP, Escuder MML, et al. 2015 Health Survey of São Paulo with Focus in Nutrition: Rationale, Design, and Procedures. Nutrients. 2018;10(2). pii: E169. 10.3390/nu10020169 PMC5852745 29389885

[21] Faludi AA, Izar MCO, Saraiva JFK, Chacra APM, Bianco HT, Afiune Neto A, et al. Atualização da Diretriz Brasileira de Dislipidemias e Prevenção da Aterosclerose – 2017. Arq Bras Cardiol. 2017;109(2 Supl 1):1-76. 10.5935/abc.20170121 28813069

[22] Matthews DR, Hosker JP, Rudenski AS, Naylor BA, Treacher DF, Turner RC. Homeostasis model assessment: insulin resistance and beta-cell function from fasting plasma glucose and insulin concentrations in man. Diabetologia. 1985;28(7):412-9. 10.1007/BF00280883 3899825

[23] Katz A, Nambi SS, Mather K, Baron AD, Follmann DA, Sullivan G, et al. Quantitative insulin sensitivity check index: a simple, accurate method for assessing insulin sensitivity in humans. J Clin Endocrinol Metab. 2000;85(7):2402-10. 10.1210/jcem.85.7.6661 10902785

[24] American Diabetes Association. Standards of Medical Care in Diabetes. Diabetes Care. 2017;40:S1-2.

[25] Stern SE, Williams K, Ferrannini E, DeFronzo RA, Bogardus C, Stern MP. Identification of individuals with insulin resistance using routine clinical measurements. Diabetes. 2005;54(2):333-9. 10.2337/diabetes.54.2.333 15677489

[26] Harttig U, Haubrock J, Knüppel S, Boeing H; EFCOVAL Consortium. The MSM program: web-based statistics package for estimating usual dietary intake using the Multiple Source Method. Eur J Clin Nutr. 2011;65 Suppl 1:S87-91. 10.1038/ejcn.2011.92 21731011

[27] Yoshida M, Booth SL, Meigs JB, Saltzman E, Jacques PF. Phylloquinone intake, insulin sensitivity, and glycemic status in men and women. Am J Clin Nutr. 2008;88(1):210-5. 10.1093/ajcn/88.1.210 PMC4457282 18614743

[28] Thijssen HH, Drittij-Reijnders MJ. Vitamin K status in human tissues: tissue-specific accumulation of phylloquinone and menaquinone-4. Br J Nutr. 1996;75(1):121-7. 10.1079/bjn19960115 8785182

[29] Stenberg LM, Nilsson E, Ljungberg O, Stenflo J, Brown MA. Synthesis of gamma-carboxylated polypeptides by alpha-cells of the pancreatic islets. Biochem Biophys Res Commun. 2001;283(2):454-9. 10.1006/bbrc.2001.4808 11327723

[30] Ohsaki Y, Shirakawa H, Hiwatashi K, Furukawa Y, Mizutani T, Komai M. Vitamin K suppresses lipopolysaccharide-induced inflammation in the rat. Biosci Biotechnol Biochem. 2006;70(4):926-32. 10.1271/bbb.70.926 16636460

[31] Shea MK, Booth SL, Massaro JM, Jacques PF, D’Agostino RB Sr, Dawson-Hughes B, et al. Vitamin K and Vitamin D Status: Associations with Inflammatory Markers in the Framingham Offspring Study. Am J Epidem. 2008;167(3):313-20. 10.1093/aje/kwm306 PMC3151653 18006902

[32] Thane CW, Wang LY, Coward WA. Plasma phylloquinone (vitamin K1) concentration and its relationship to intake in British adults aged 19-64 years. Br J Nutr. 2006;96(6):1116-24. 10.1017/bjn20061972 17181887

[33] Takeuchi Y, Suzawa M, Fukumoto S, Fujita T. Vitamin K(2) inhibits adipogenesis, osteoclastogenesis, and ODF/RANK ligand expression in murine bone marrow cell cultures. Bone. 2000;27(6):769-76. 10.1016/s8756-3282(00)00396-3 11113387

[34] Pereira JC, Barreto SM, Passos VMA. The Profile of Cardiovascular Health of Elderly Brazilian People Needs to Improve: a Population-Based Study. Arq Bras Cardiol. 2008;91(1):1-10. 10.1590/s0066-782x2008001300001 18660938

[35] World Health Organization (2016). Global Report on Diabetes. Accessed in: April 2, 2018.

